# CDKN1A as a target of senescence in heart failure: insights from a multiomics study

**DOI:** 10.3389/fphar.2024.1446300

**Published:** 2024-10-23

**Authors:** Rutao Bian, Li Zhang, Dongyu Li, Xuegong Xu

**Affiliations:** ^1^ Department of Cardiology, Zhengzhou Hospital of Traditional Chinese Medicine, Zhengzhou, Henan, China; ^2^ The Affiliated Zhengzhou Hospital of Traditional Chinese Medicine, Henan University of Chinese Medicine, Zhengzhou, Henan, China

**Keywords:** heart failure, Mendelian randomization, cardiomyocyte, senescence, omics analysis

## Abstract

**Background:**

Cardiomyocyte senescence plays a crucial role as a pathological mechanism in heart failure (HF). However, the exact triggering factors and underlying causes of HF onset and progression are still not fully understood.

**Objectives:**

By integrating multi-omics data, this study aimed to determine the genetic associations between cardiomyocyte and HF using cell senescence-related genes (SRGs).

**Methods:**

The study utilized the CellAge database and the SenMayo dataset, combined with high-resolution single-cell RNA sequencing (scRNA-seq) data, to identify SRG and examine differences in cardiac cell expression. To explore the causal relationship with HF using Mendelian Randomization (MR). Genetic variations influencing gene expression, DNA methylation, and protein expression (cis-eQTL, cis-mQTL, and cis-pQTL) were analyzed using the two-sample MR (TSMR) and summary-data-based MR (SMR). Additionally, Bayesian colocalization analysis, germline genetic variation, and bulk RNA data were employed to strengthen the reliability of the results. The application potential of therapeutic targets is ultimately assessed by evaluating their druggability.

**Results:**

The expression of 39 SRGs in cardiomyocytes was identified. In the discovery set revealed that *CDKN1A* (OR = 1.09, 95% confidence interval (CI) 1.02–1.15, FDR = 0.048) could be causally related to HF, and the results are also replicated in the validation set (OR = 1.20, 95% confidence interval (CI) 1.10–1.30, FDR <0.0001). Based on the SMR method, *CDKN1A* was confirmed as a candidate pathogenic gene for HF, and its methylation (cg03714916, cg08179530) was associated with HF risk loci. The result is validated by Bayesian colocalization analysis, genetic variations, and bulk RNA data. The druggability analysis identified two potential therapeutic drugs.

**Conclusion:**

Based on multi-omics data, this study uncovered the reciprocal regulation of cardiomyocyte senescence through *CDKN1*A, providing potential targets for HF drug development.

## Introduction

Heart failure (HF) is a complex syndrome characterized by decreased filling or poor ejection, along with symptoms such as dyspnea and fatigue (Baman and Ahmad, 2020). The role of aging as a major risk factor for cardiovascular disease is often overlooked (North and Sinclair, 2012). Even in the absence of systemic risk factors like smoking, dyslipidemia, hypertension, and diabetes, intrinsic cardiac aging can lead to a decline in cardiac structure and function in the elderly. Epigenetic changes are implicated in various age-related cardiac diseases, such as ischemic heart disease, which might make the heart more susceptible to aging. The accelerated aging of the population increases the risk of HF, particularly in individuals with chronic diseases, which imposes a significant economic burden on the public health system (Shahim et al., 2023). Therefore, understanding the triggers of cardiac aging and identifying key molecular targets that contribute to it is essential.

Several mechanisms contribute to senescence, including increased oxidative stress, stem cell depletion, altered cell communication, reduced genomic stability, shortening of telomeres, epigenetic changes, disrupted protein homeostasis, impaired nutrient metabolism, and mitochondrial dysfunction, which lead to changes at the molecular, cellular, tissue, and organ levels (López-Otín et al., 2013; Yin et al., 2022). Consequently, hypertrophy, fibrosis, protein misfolding, mitochondrial dysfunction, and an increase in sympathetic nerve activity are observed (Steenman and Lande, 2017; Evangelou et al., 2023). Senescence-related genes (SRGs) in human tissues are linked to accelerated aging. Furthermore, these SRGs are not restricted to specific tissues, indicating that their expression varies between different organs and cell types, which results in distinct biological functions (Hernandez-Segura et al., 2018). Cardiomyocytes, which make up 30%–40% of the total cardiac cells, undergo morphological and functional changes with age. Studies have shown that elderly hearts contain a significant number of senescent cells, which may be responsible for structural and functional changes, such as hypertrophy of the left ventricle, decreased diastolic function, and pathological changes including myocardial fibrosis, extracellular matrix remodeling, and conduction block (Hu et al., 2022).

Meanwhile, some studies have partially revealed the connection between aging-related gene regulation and HF. Currently, traditional observational studies have identified associations between specific cell senescence-related genes and HF risk. For example, mild to moderate expression of Sirt1 can delay cardiac aging (Alcendor et al., 2007), and vaccine therapies targeting Igfbp7 may help prevent the development of HF (Xie et al., 2023; Katoh et al., 2024). However, the underlying mechanisms involving epigenetic changes remain unclear. Genome-wide association studies (GWAS) have uncovered intricate details of the pathophysiology of complex diseases, providing important insights. These extensive studies have identified numerous disease-related genetic loci, further enhancing our understanding of the critical role genetic factors play in disease etiology.

Mendelian randomization (MR) and Summary data-based Mendelian randomization (SMR) are utilized to assess causal links or pleiotropy between genetic and complex traits. MR employs genetic variants as instrumental variables (IVs) to explore potential causal connections between exposures over a lifetime and their outcomes (Davey Smith and Hemani, 2014). Additionally, research on quantitative trait loci (QTL) has been crucial in determining which genes are influenced by GWAS by analyzing how gene sequences correlate with gene expression. SMR seeks to integrate GWAS data with molecular trait data to accurately identify disease susceptibility genes (Zhu et al., 2016). HEIDI tests are employed to differentiate between widespread linkage disequilibrium (LD) and possible causal associations (Zhu et al., 2016; Lu et al., 2017). GWAS connects single nucleotide polymorphisms (SNPs) with gene expression and methylation profiles to uncover genetic links to various traits. SNPs are highly effective in pinpointing genetic risk loci for complex diseases such as rheumatoid arthritis, schizophrenia, and Alzheimer’s disease, emphasizing their significance in genetic research (Steinberg et al., 2021; Lee et al., 2022; Zhou et al., 2024). This study uses ScRNA-seq, bulk RNA, and MR to identify risk loci for HF cardiomyocyte senescence, providing a scientific rationale to improve the prevention and treatment of HF.

## Materials and methods

### Study design

**FIGURE 1:**
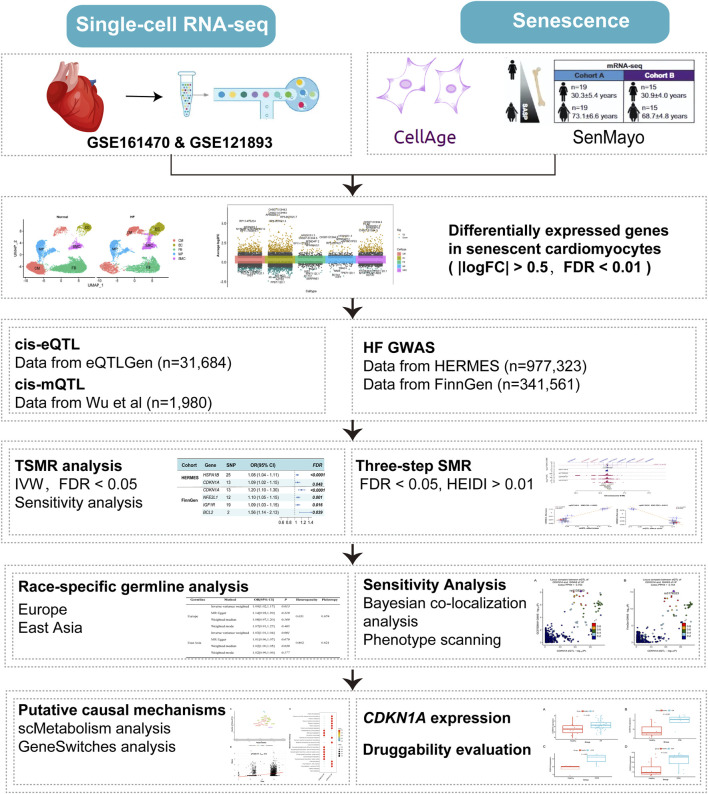
Flowchart of the analyses performed. HF, Heart failure; TSMR, two-sample MR, SMR, summary-data-based MR, IVW, inverse-variance weighted, FDR, false discovery rate, HEIDI, heterogeneity in dependent instruments.

An overview of the study’s workflow can be found in Figure 1. We obtained 307 genes from the CellAge database (Chatsirisupachai et al., 2019; Avelar et al., 2020) and 125 genes from the SenMayo set defined by Saul et al. (2022). With duplicate genes removed, 413 senescence-related genes were listed (Additional file 1: Supplementary Table S1). Initially, differential gene analysis in cardiomyocytes was performed using ScRNA-seq dataset. Two-sample MR (TSMR) and SMR (Zhu et al., 2016) analysis were used to identify potential risk loci for HF using the cardiomyocyte SRGs. In addition, bulk RNA-seq of various types of HF was performed to assess the reliability of the findings. This study followed the STROBE-MR (Skrivankova et al., 2021) (Strengthening the reporting of observational studies in epidemiology using mendelian randomization) guidelines and the STROBE-MR reporting checklist (Additional file 2: Supplementary Datasheet S1).

### Ethics approval

In this study, data were obtained from public databases approved for original research, thereby avoiding the need for additional ethical approval.

### scRNA-seq data analysis

ScRNA-seq datasets GSE161470 (Luo et al., 2021) and GSE121893 (Wang et al., 2020) were retrieved from the Gene Expression Omnibus (GEO) database (Additional file 1: Supplementary Table S2), and were analyzed using the “Seurat” package (Stuart et al., 2019). Single-cell objects were constructed based on the expression of each gene set in at least any three cells. There was no filtering for cells expressing fewer than 200 or more than 6,000 genes in paired samples. The feature “FindVariableFeatures” to identify highly variable genes. After filtering the data using the “harmony” package and principal component analysis (PCA), 17 clusters were identified. Annotation of representative cell types was performed using marker genes (Wang et al., 2020). A total of 23,053 cells were annotated with the following cell types based on their marker genes: cardiomyocytes (CM), endothelial cells (EC), fibroblasts (FB), macrophages (MP), and smooth muscle cells (SMC). In this study, “FindAllMarkers” (min. pct = 0.25, logfc = 0.50). To quantify differential gene expression among subgroups, we conducted a statistical test using a false discovery rate (FDR) threshold of less than 0.05, applying the Benjamini–Hochberg (BH) method for calculation (Benjamini and Hochberg, 2018). Comparing metabolic spectrums across different cell types was achieved via the scMetabolism (Wu et al., 2022) tool. Using GeneSwitches (Cao et al., 2020), gene transcription and functional events during HF activation are identified, revealing the genes that act as switches between cell states. Upregulated switch genes are those with a pseudotemporal correlation (*R* ^2^ > 0), while silent switch genes with a pseudotemporal correlation (*R* ^2^ < 0) are considered downregulated genes. The acceleration process correlates more closely with switch genes when the pseudotemporal correlation is higher.

### Bulk RNA-seq data analysis

GEO data were used to retrieve bulk RNA information (Additional file 1: Supplementary Table S2). The dataset GSE116250 (Sweet et al., 2018) consists of 64 samples, including 37 cases of dilated cardiomyopathy, 13 cases of ischemic cardiomyopathy, and 14 non-HF specimens. The dataset GSE203160 (Wang et al., 2022) includes 15 samples, 8 of which are ischemic cardiomyopathy cases and 7 of which are non-HF cases. The GSE89714 dataset contains 9 samples, including 5 cases of hypertrophic cardiomyopathy and 4 non-HF cases. The GSE206803 (Chen et al., 2023) study included 22 human induced pluripotent stem cell-derived cardiomyocyte samples, 11 cases of amyloid cardiomyopathy, and 11 control samples. Using a linear regression model and adjusting for covariates like age and gender (if applicable), we could identify differences between non-HF individuals and specific categories of HF patients.

### GWAS data

HF data from the FinnGen study (Kurki et al., 2023) and the Heart Failure Molecular Epidemiology for Therapeutic Targets consortium (HERMES) (Shah et al., 2020) were used in this study (Additional file 1: Supplementary Table S2). The FinnGen study consists of 23,622 cases and 317,939 controls, while the HERMES study consists of 47,309 cases and 930,014 controls.

This data is sourced from the eQTLGen Consortium (https://www.eqtlgen.org/cis-eqtls.html) (Võsa et al., 2021), which covers 37 datasets and 31,684 individuals. To mitigate the likelihood of horizontal pleiotropy, cis-eQTLs were used as instrumental variables. Summary cis-mQTL data from two European cohorts (n = 1980) are used to select cis-eQTL instruments linked to DNA methylation (DNAm)-related genes (Wu et al., 2018). The protein quantitative trait loci (pQTL) data originated from the UKB-PPP (http://ukb-ppp.gwas.eu) (Sun et al., 2023), a joint initiative that conducted an in-depth analysis of plasma proteomic profiles across 54,219 UKB participants with germline genetic variation. This research delivered an extensive mapping of cis-pQTL involving 2,923 distinct proteins.

### IVs selection

IVs related to single nucleotide polymorphisms (SNPs) of the target gene were extracted from the dataset. The MR analysis aimed to investigate the causal relationship between SRGs and the risk of HF, utilizing these SNPs as IVs. SNPs with significance threshold <5.0E-08, minor allele frequency (MAF) > 0.01, SNP allele frequency difference <0.2, and maximum allele frequency difference ratio <0.05, were identified. Only robust IVs with F-statistics exceeding 10 were retained. To validate the IVs, three key assumptions were imposed: 1) a strong correlation between genetic instruments and the exposure, 2) no correlation with potential confounders, and 3) no correlation with confounders influencing the exposure-outcome relationship.

### SMR and colocalization analysis

In SMR, gene expression, DNAm, and exposure outcomes are analyzed for their pleiotropic associations. The heterogeneity in dependent instruments (HEIDI) test to evaluate pleiotropy (Zhu et al., 2016; Lu et al., 2017). An SMR analysis was performed using SNPs as genetic IVs, cis-eQTLs and DNAms as exposure factors, and HF as the outcome. An analysis of sensitivity was conducted to evaluate the robustness of the results. To establish the final causal relationship, the following criteria must be met in the three-step SMR (Zou et al., 2023): 1) FDR <0.05, 2) HEIDI >0.01, and 3) eQTL and mQTL should correspond to the same gene symbol.

Bayesian colocalization analyses test whether GWAS summary data and eQTL share causal variants. It assesses the posterior probability of each hypothesis based on five hypotheses (H0, no association with either GWAS or QTL at the locus; H1, the association only with GWAS; H2, the association only with QTL; H3, the association only with GWAS and QTL but not colocalized; H4, colocalization of GWAS and QTL). A colocalization analysis was performed on all SNPs within 100 kb of the top SNP of the probe. Several loci with PPH4 ≥ 0.5 appear to align with the colocalization suggested by PPH4 ≥ 0.8 (Dobbyn et al., 2018).

### Druggability analysis

We utilized DGldb (Freshour et al., 2021) to evaluate the druggability of potential target proteins. These resources provide comprehensive insights into drug-target interactions and aid in determining the therapeutic viability of target genes.

### Statistical analysis

To calculate TSMR estimates for individual SNPs, we used the Wald ratio method (Teumer, 2018), the inverse-variance weighted (IVW) model (Burgess et al., 2016), which utilizes multiple IVs for genes. To minimize potential omissions of target genes, FDR <0.05 was established (Zhang et al., 2022). MR-Egger (Bowden et al., 2015) intercepts and Cochran’s Q were used to measure horizontal pleiotropy and heterogeneity. PhenoScanner (Kamat et al., 2019) was also used to explore associations between identified QTLs and other traits. The leave-one-out analysis was utilized to identify potential outliers that could introduce significant bias into the results. Subsequently, these outliers were excluded, and the MR analyses were re-performed.

The SMR analysis and HEIDI testing were conducted using version 1.3.1 of the SMR software available (https://yanglab.westlake.edu.cn/software/smr/#Download). TSMR analysis was performed utilizing the “TwoSampleMR (version 4.2.2)” package within R software (version 0.5.6). Colocalization analysis was carried out using the “coloc (version 3.3.0)” R package. Differential analysis of datasets utilized the “Limma (version 1.2.6)” package, while result visualization was achieved using the “forestploter (version 3.0.1)” package.

## Results

### The selection of differentially expressed genes in cardiomyocyte senescence

**FIGURE 2:**
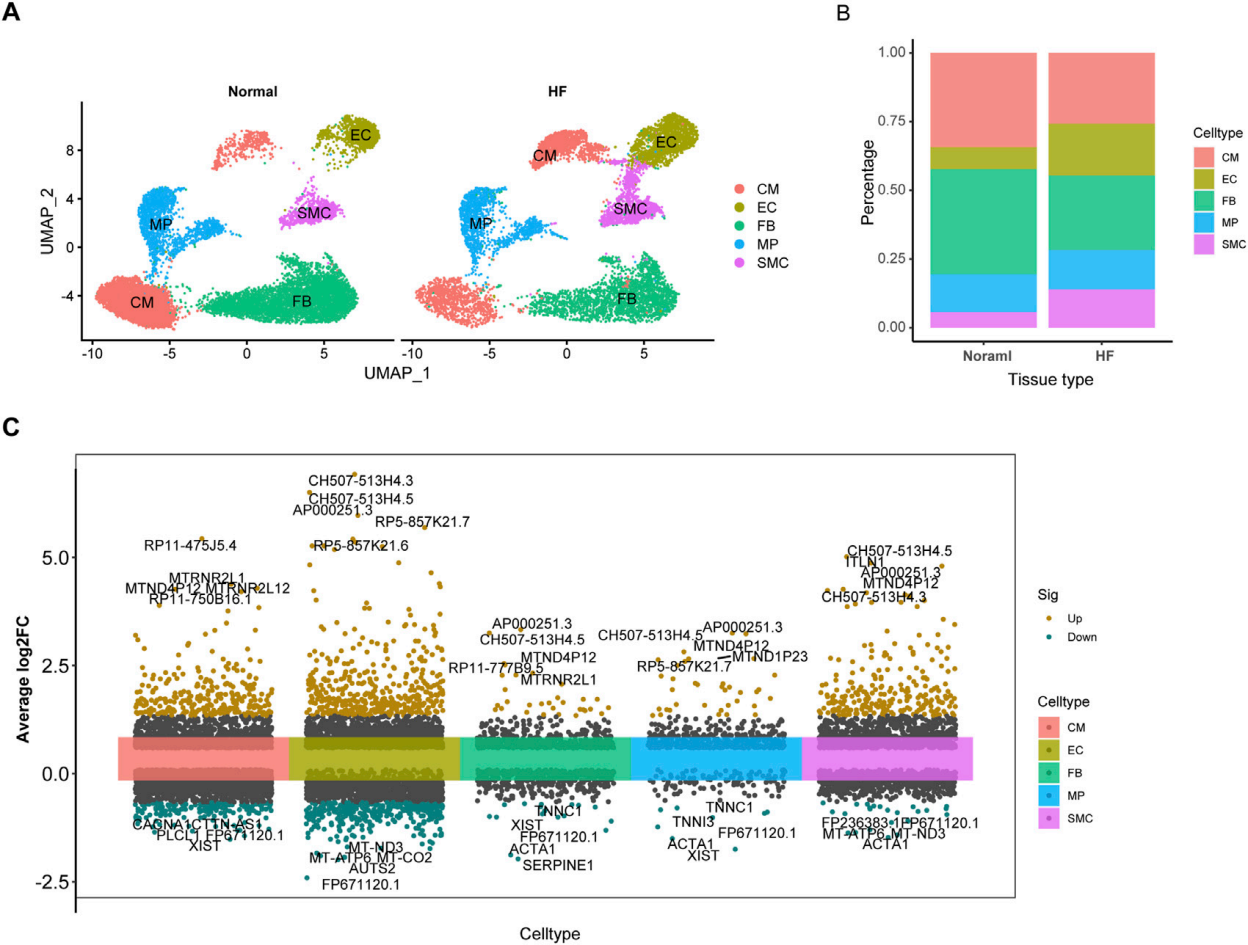
The expression of SRGs is revealed by ScRNA-seq analysis. **(A)**, Defining cardiac tissue cells. **(B)**, Histogram displaying distinct subtypes of myocardial cells based on categorical variables. **(C)**, Visualization of differential gene expression across single-cell subtypes.

A total of 23,053 cells passed the quality control and were included in the analysis. A total of 5 cell subtypes were identified (Figures 2A, B), including CM, EC, FB, MP, and SMC, “FindVariableFeatures” identifies highly variable genes in cell subtypes (Figure 2C, Additional file 1: Supplementary Table S3). In addition, using a |logFC| > 0.5 and FDR <0.01, we detected 39 differentially expressed cardiomyocyte senescence genes associated with HF (Additional file 1: Supplementary Table S4).

### TSMR analysis of cardiomyocyte aging

**FIGURE 3:**
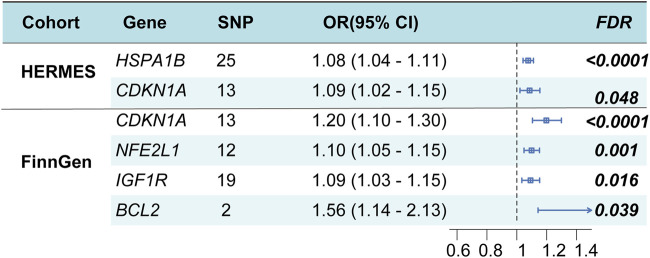
Cardiomyocyte senescence and HF risk estimation by TSMR analysis. OR, odds ratio; CI, confidence interval.

After eliminating outliers for the multiple regression analysis, the F statistics of all SNPs surpassed 10, suggesting that these SNPs are appropriate for utilization as robust instrumental factors. The findings suggest that heat shock protein family A member 1B (*HSPA1B*), cyclin-dependent kinase inhibitor 1A (*CDKN1A*) may serve as potential genes, and the overall causal effects of cis-eQTL of all genes on HF were summarized (Figure 3; Additional file 1: Supplementary Tables S5, S8, S9). The MR-Egger method was utilized to assess the sensitivity and directional pleiotropy of different genes, and the Egger intercept results of MR-Egger indicated that all variable *P* > 0.05, suggesting no significant pleiotropy was present (Additional file 1: Supplementary Table S7). Additionally, the leave-one-out test results demonstrated that, upon removing each SNP in turn, the remaining SNPs yielded similar analysis results to the inclusion of all SNPs, with no SNP significantly influencing the estimated causal values. Furthermore, the FinnGen study validated *CDKN1A* as a potential risk locus for HF (Figure 3; Additional file 1: Supplementary Table S6).

### SMR analysis of senescence genome-wide cis-eQTLs and HF

**FIGURE 4:**
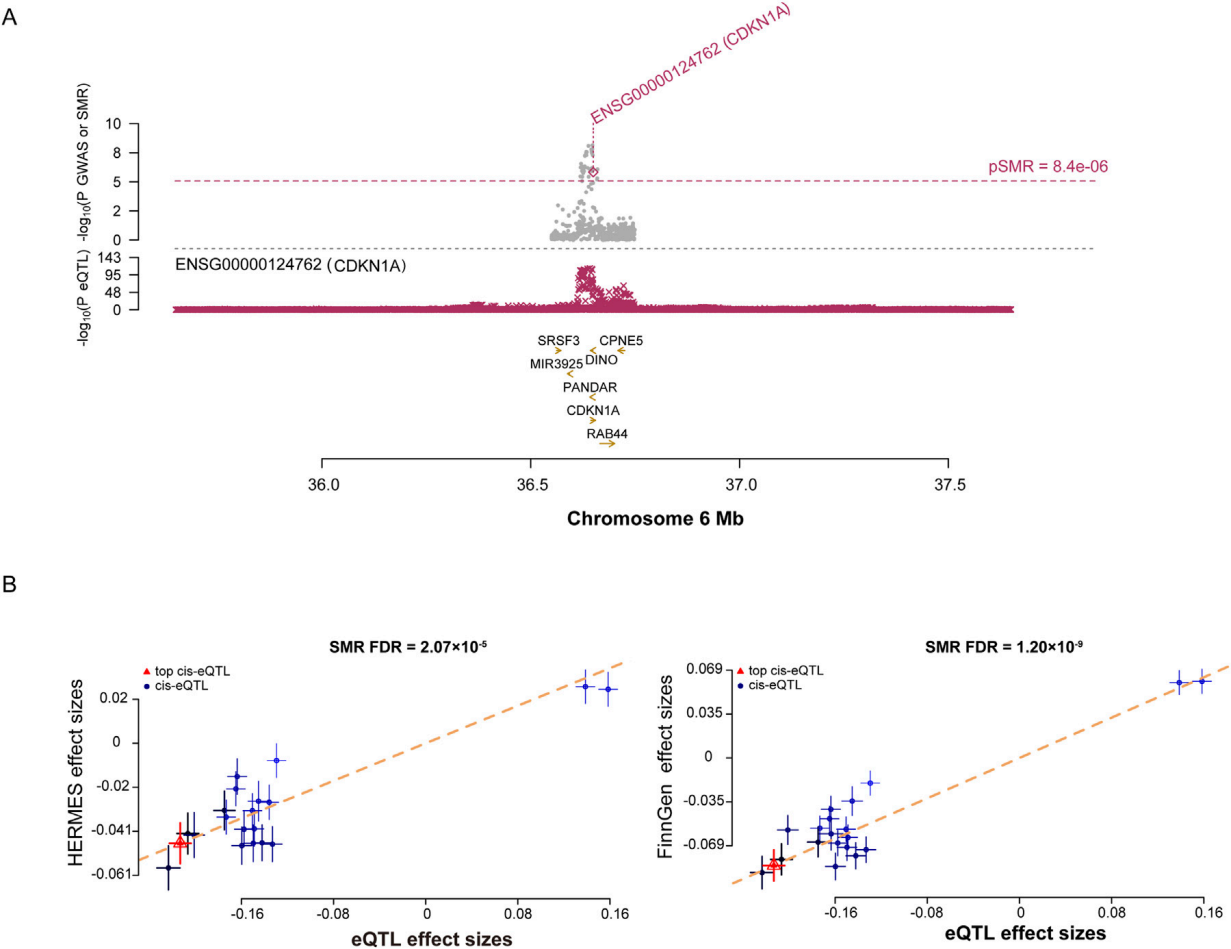
SMR analysis prioritized putative causal SRGs in HF. **(A)**, plots displaying the consistent genetic effects from HF GWAS in the cis-eQTL region near *CDKN1A*. **(B)**, SMR results indicate significant causal relationships between *CDKN1A* gene expressions and HF.

The SMR results of 2055 SNPs representing SRG expressions were associated with the experience of HF (Additional file 1: Supplementary Table S10). As a result of test correction, only *CDKN1A* showed a strong association (Figure 4, FDR = 2.07E-05), whereas the subsequent HEIDI test excluded pleiotropy. Additionally, the *CDKN1A* and HF have been linked in the FinnGen cohort (Additional file 1: Supplementary Table S10, FDR = 1.20E-09). The findings of our study suggest an increased risk of HF in people with high *CDKN1A* expression.

### SMR analysis of SRGs genome-wide cis-mQTLs and HF

**FIGURE 5:**
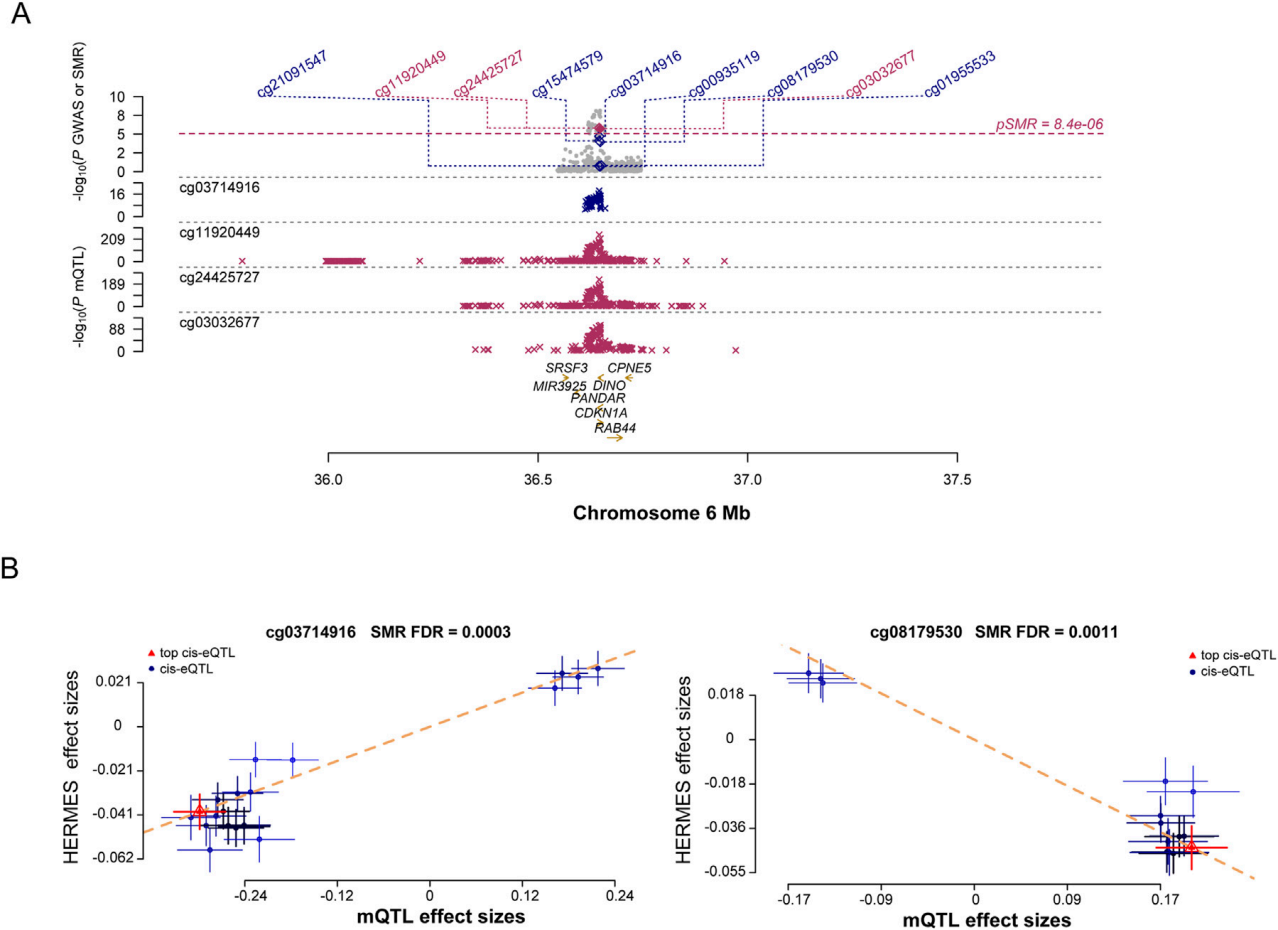
Results of SNPs and SMR associations across cis-mQTL and HF GWAS. **(A)**, Locus zoom plots illustrating the consistent genetic effects from HF GWAS in cis-mQTL near *CDKN1A*. **(B)**, SMR indicating significant causal relationships between *CDKN1A* gene expressions and HF.

The causal relationship between DNAm in SRGs and HF was examined. According to SMR analysis of mQTL data, 195 CpG sites were identified (Figure 5A; Additional file 1: Supplementary Table S11). These sites corresponded to nine genes associated with HF. According to the HEIDI test, *CDKN1A* exhibited six independent sites, while *EGR1*, *ADCY5*, and *MAP3K5* each exhibited one. In the FinnGen cohort, only cg03714916 (FDR = 3.54E-06, Additional file 1: Supplementary Table S12) and cg08179530 (FDR = 5.96E-05, Additional file 1: Supplementary Table S13) passed validation. There was a positive correlation between *CDKN1A* expression and HF onset at site cg03714916 (Figure 5B). In contrast, a negative correlation was found between *CDKN1A* expression and HF onset at site cg08179530 (Figure 5B), indicating an association between higher *CDKN1A* expression and HF onset. Therefore, lower levels of DNAm in the *CDKN1A* enhancer region may stimulate gene expression, thereby increasing the risk of HF.

As a result of investigating the relationship between cis-mQTL and cis-eQTL, according to the FDR and HEIDI test, we observed significant interactions between 37 sites of 17 genes (Additional file 1: Supplementary Table S14). Our analysis of the results of the previous two steps found a significant association between cg08179530 and a reduced risk of HF (P_SMR_ = 1.47E-08) and a significant association between cg15474579 and an increased risk of HF (P_SMR_ = 5.48E-36). A three-step SMR analysis showed that the SNP signals and *CDKN1A* in the HF GWAS, cis-mQTL, and cis-eQTL data studies were highly significant.

### Sensitivity analysis

**FIGURE 6:**
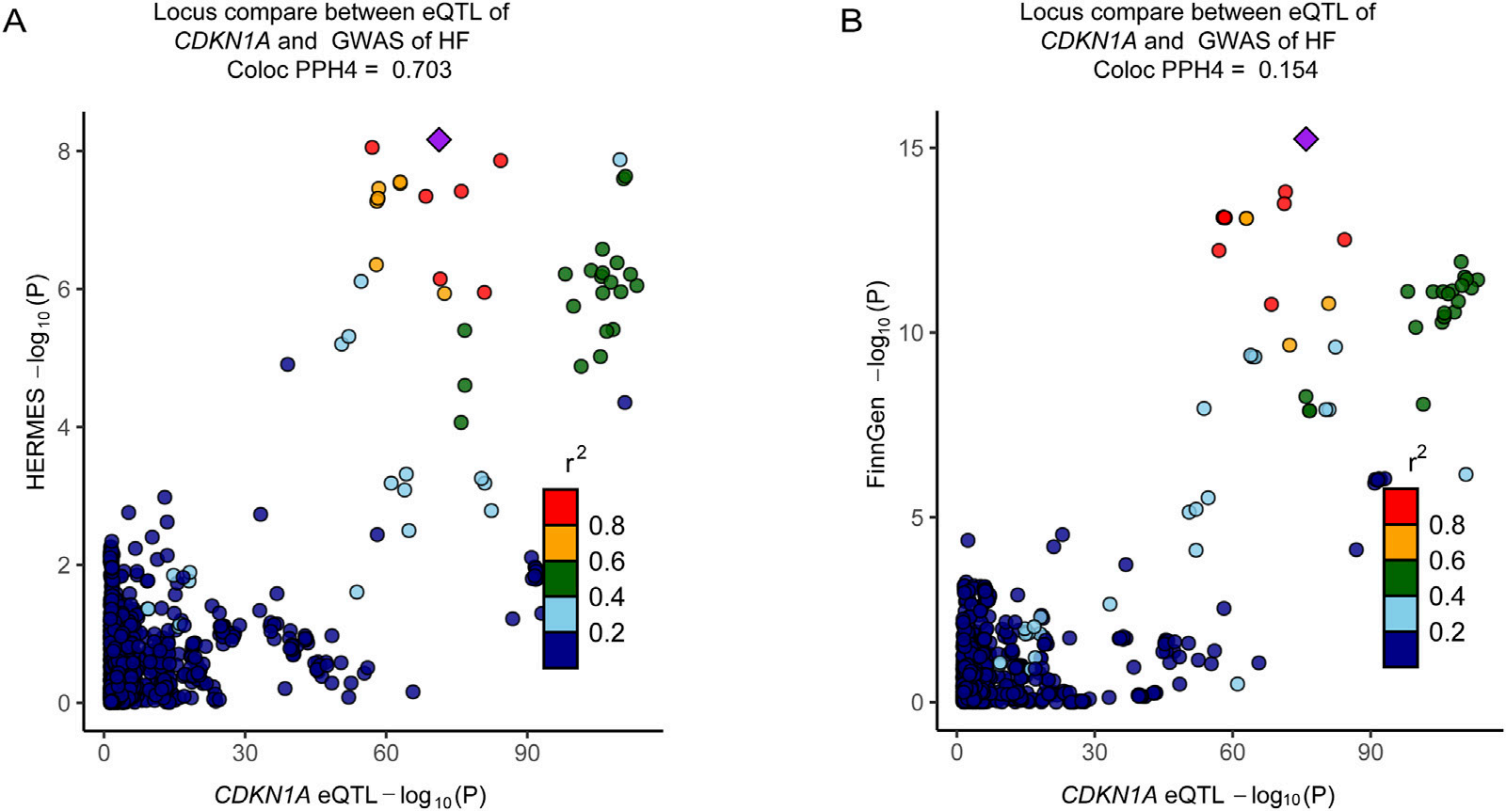
Colocalization analyses prioritized intestinal causal CDKN1A and interactions with HF. **(A)**, Colocalization analysis of *CDKN1A* locus genes with HF (HERMES, PPH4 = 0.703). **(B)**, Colocalization analysis of the genes of *CDKN1A* locus with the genes of HF (FinnGen cohort, PPH4 = 0.154). The *r* ^2^ value indicates the LD between the variants and the top SNPs.

A colocalization analysis was conducted to assess the influence of linkage disequilibrium. A posterior probability (PP.H4) of shared causality between trans-gene expression and HF greater than 0.50 suggests that HF GWAS and eQTL are colocalized. Bayesian colocalization results indicate that *CDKN1A* and HF share genetic variation in the Consortium dataset (Figure 6A; Additional file 1: Supplementary Table S15). However, *CDKN1A* and HF shared no genetic variation in the FinnGen cohort (Figure 6B). In addition, phenotype scanning has shown that *CDKN1A* is associated with trunk fat-free mass, trunk predicted mass, and triglycerides, while total cholesterol is associated with atopic dermatitis (Additional file 1: Supplementary Table S16).

### Race-specific germline analysis

To evaluate the relationship between germline genetic variation in the *CDKN1A* gene and HF, TSMR analysis was performed on *CDKN1A* cis-pQTL among distinct racial groups. This analysis compared European and East Asian populations and identified significant disparities in the associations (Table 1).

**TABLE 1:** Race-specific germline analysis.

Germline	Method	OR [95% CI]	*P*	Heterogeneity	Pleiotropy
Europe	Inverse variance weighted	1.09 [1.02,1.17]	*0.013*	0.631	0.674
	MR Egger	1.14 [0.93,1.39]	*0.210*		
	Weighted median	1.08 [0.97,1.20]	*0.160*		
	Weighted mode	1.07 [0.91,1.27]	*0.405*		
East Asia	Inverse variance weighted	1.03 [1.01,1.04]	*0.001*	0.802	0.621
	MR Egger	1.01 [0.96,1.07]	*0.670*		
	Weighted median	1.02 [1.00,1.05]	*0.030*		
	Weighted mode	1.02 [0.99,1.06]	*0.177*		

OR, odds ratio; CI, confidence interval.

### Assessing CDKN1A potential role in cardiomyocyte senescence in HF

**FIGURE 7:**
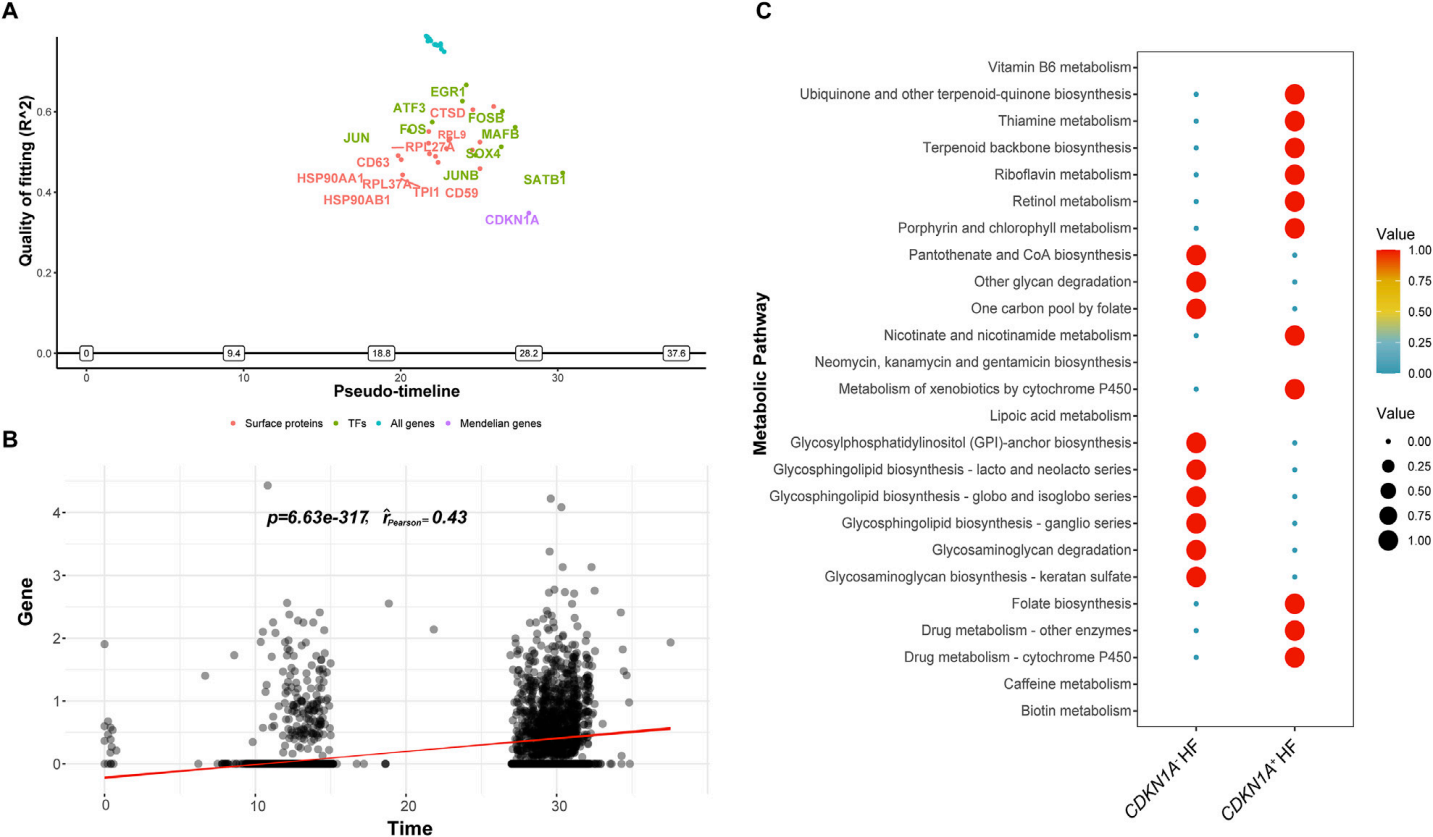
The ScMetabolism and GeneSwitches analyses from HF. **(A)**, Diagram of the top fitting switching genes from various sets of known proteins along the pseudotime. A positive or negative sign on the *y*-axis indicates upregulation or downregulation as defined by McFadden’s pseudo *R* ^2^. “Transcription factors (TFs)” refers to transcription factors. **(B)**, *CDKN1A* expression escalating over time is demonstrated by pseudotime correlation analysis. **(C)**, The bubble plot illustrates the two main metabolic pathways for *CDKN1A* ^+^ and *CDKN1A* ^−^.

A pseudotime analysis was performed to investigate the late-switching events of *CDKN1A* in HF. The expression of *HSP90AA1, HSP90AB1, JUN, CD63*, and *ATF3* was upregulated before *CDKN1A*, and *SATB1* expression increased with the increase in *CDKN1A* expression. *EGR1* displayed the best McFadden’s Pseudo *R* ^2^ fit quality and may regulate cardiomyocyte senescence through *CDKN1A* expression (Figures 7A, B).

For the investigation of metabolic pattern dominance in cardiomyocyte with *CDKN1A* (*CDKN1A* ^+^ and *CDKN1A* ^−^), we used ScMetabolism to score metabolic pathways quantitatively. A study revealed that cardiomyocytes with *CDKN1A* ^+^ showed increased biosynthesis of ubiquinone, other terpenoid-quinones, thiamine, riboflavin, nicotinate, and nicotinamide. In contrast, *CDKN1A* ^−^ cardiomyocytes produced more pantothenate and CoA, degraded other glycans, and synthesized glycosphingolipids (Figure 7C). *CDKN1A* may mediate a variety of biological processes in HF cardiomyocytes.

### Meta-analysis of CDKN1A in HF

**FIGURE 8:**
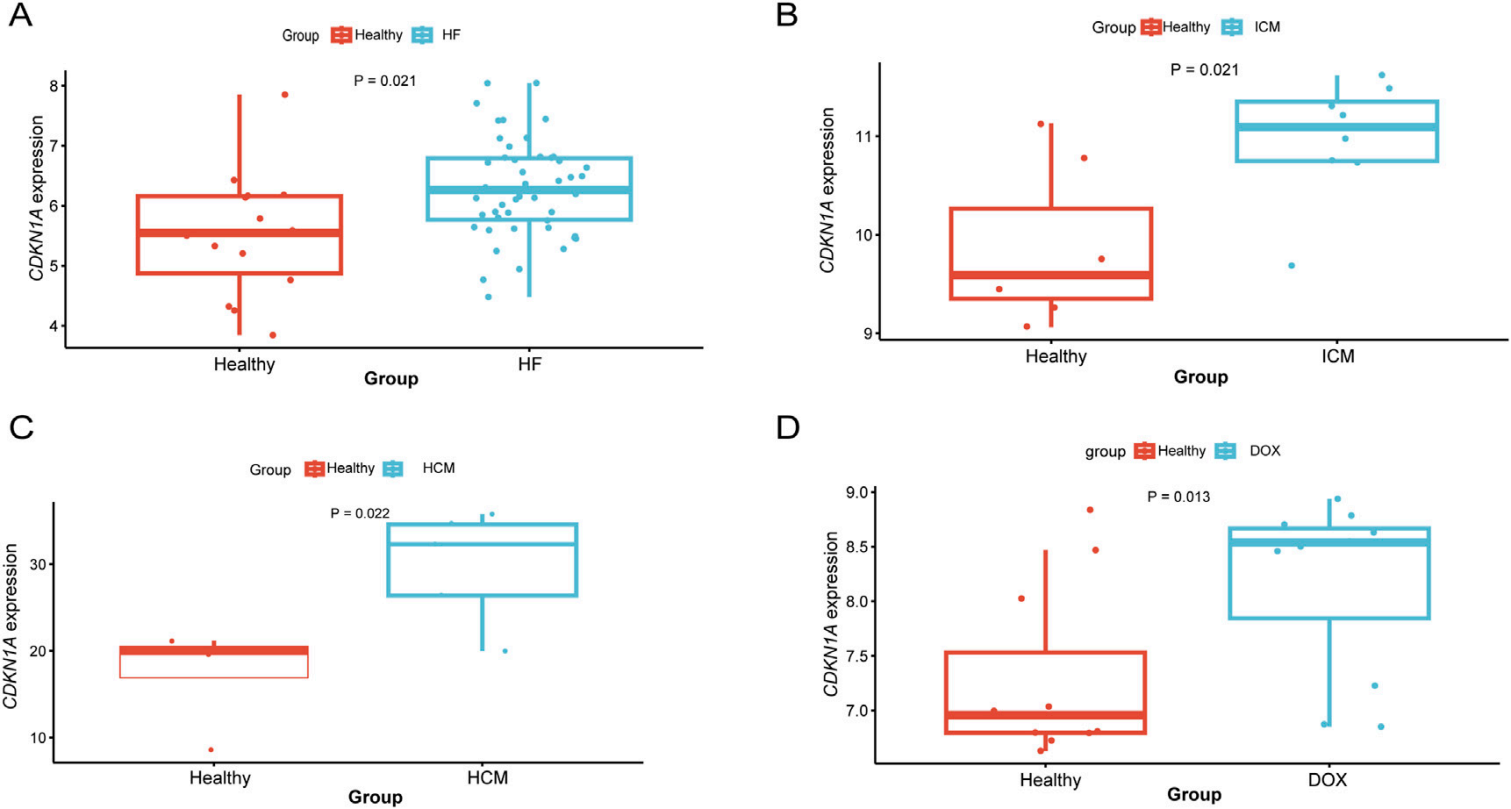
Differential expression analysis of *CDKN1A* in different types of HF. **(A)**, HF mixed type sample (GSE116250). **(B)**, ICM (GSE203160). **(C)**, hypertrophic cardiomyopathy (GSE89714). **(D)**, Doxorubicin (GSE206803).

To enhance the reliability of this study, data from a variety of types of HF were analyzed. A differential analysis revealed higher expression in mixed heart disease (GSE116250) (Figure 8A). Increased expression in ischemic heart disease (GSE203160), hypertrophic heart disease (GSE89714), and anthracycline heart disease (GSE206803) (Figures 8B–D). After adjusting for gender and age effects by linear regression, the meta-analysis indicated that *CDKN1A* is a significant risk factor for HF (Additional file 1: Supplementary Table S17). There is the evidence that *CDKN1A* may be an effective target for the treatment of HF.

### Druggability evaluation

In the assessment of drug availability, the approved drugs sodium salicylate and dicoumarol were identified as potential regulators of CDKN1A. Sodium salicylate inhibits cyclooxygenase, reducing prostaglandin production and offering anti-inflammatory, anti-rheumatic, and analgesic effects. Dicoumarol acts as an anticoagulant, frequently used in the prevention and treatment of thromboembolic disorders.

## Discussion

The degeneration of heart function and structure resulting from aging is primarily characterized by symptoms such as myocardial hypertrophy, myocardial fibrosis, and HF. Cell senescence and death result in the cessation of fibrillation and proliferation in mature cardiomyocytes. The slowing down of cell cycles is not the most common feature of cardiomyocyte senescence, and they typically experience some degree of functional decline with aging (Li et al., 2020). The TSMR analysis initially suggested HSPA1B, CDKN1A as potential risk loci. However, the FinnGen study confirmed only CDKN1A as a potential risk locus for HF. To further validate these findings, Bayesian colocalization analysis, genetic variation in the germline, and extensive RNA data were utilized. The study also investigated the connections between specific SNPs and CpG sites and the occurrence of HF, as well as the relationship between cellular characteristics and CDKN1A expression. Strong evidence indicates that the CDKN1A gene locus, along with its methylation status and expression levels, plays a role in HF pathogenesis. The druggability analysis identified sodium salicylate and dicoumarol as potential therapeutic drugs.

HSPA1B is an important component of the HSP70 family, acts as a molecular chaperone essential for cellular stress responses, protein folding, and maintaining protein stability. Research indicates that higher levels of HSPA1B can notably decrease myocardial cell death during ischemia-reperfusion injury (Liu et al., 2021; Tao et al., 2021). Additionally, various medications have been identified that can indirectly influence HSPA1B expression and activity, showing efficacy in heart failure and related diseases (Gombos et al., 2008). *CDKN1A* is a cyclin-dependent kinase inhibitor, has been known to regulate the cell cycle process (López-Domínguez et al., 2021). Researchers have discovered that HF patients have an increased expression of *CDKN1A*. A crucial finding of our study confirms an increase in the expression level of *CDKN1A* in HF samples, similar to the findings published elsewhere (Zheng et al., 2023). Studies have indicated that *CDKN1A* may promote coronary heart disease by promoting chronic inflammation and sustained inflammatory states (Zhang et al., 2019). It has been reported that *CDKN1A* plays a significant role in inflammatory heart diseases because it is highly expressed in cardiomyocytes (Huang et al., 2020). A possible link between DNAm, and the risk of HF is also revealed by our findings that DNAm in enhancer regions negatively regulates *CDKN1A* expression. There is a genetic variation near the *CDKN1A* gene associated with HF in previous GWAS studies (Shah et al., 2020), but whether this gene is causally related to the illness remains unclear. DNAm may be involved in genetic variation regulating gene expression in HF, which impacts its pathogenesis. It has been reported that *EGR1* regulates *CDKN1A* expression, resulting in the senescence of cancer cells (Carvalho et al., 2019). More research needs to be conducted to find out whether it plays a role in HF.

*CDKN1A* mechanism is complex across a wide range of cell types and stimulus environments. A number of studies have shown that *CDKN1A* ^−^ positive senescent cells exhibit a clear convergence, initially displaying high levels of *CDKN1A* due to damage, followed by a subsequent decrease (Bloom et al., 2023). Furthermore, the function of *CDKN1A* is influenced by the degree of DNA damage in the cell. When DNA damage is low, *CDKN1A* expression increases, slowing the cell division cycle and preventing apoptosis. In contrast, with higher levels of DNA damage, *CDKN1A* expression decreases, leading to apoptosis (Karimian et al., 2016). Additionally, our study observed that HF cardiomyocytes with *CDKN1A* ^+^ show elevated levels of ubiquinone and other terpenoid-quinone biosynthesis, thiamine metabolism, and nicotinate and nicotinamide metabolism, suggesting metabolic discrepancies among HF cardiomyocytes with different *CDKN1A* genotypes. In addition to regulating fundamental processes such as cell cycle progression, apoptosis, and transcription, *CDKN1A* could also be responsible for the variation in phenotypes (Cazzalini et al., 2010). Biomarkers related to cardiac aging are a major focus in cardiovascular disease research. Compared to tissue markers and MRI (Salih et al., 2023), blood biomarkers provide advantages like simpler collection, reduced invasiveness, and the capacity for ongoing monitoring. The loci identified in this study shed new light on potential non-invasive biomarkers linked to HF. Current investigations into CDKN1A inhibitors for cancer therapies indicate promising possibilities for cardiovascular applications, which merit further investigation. More research is required to establish the safety and efficacy of these treatments in relation to HF.

Based on multi-omics data, genetic IVs, and causal inference analysis methods, this study offers significant advantages in dissecting GWAS signals and prioritizing gene expression and methylation. In addition, sensitivity tests were carried out to evaluate the robustness of the results and minimize potential biases due to pleiotropic effects. However, the study has certain limitations. The summary statistics for eQTL, mQTL, and pQTL come from varied sources, and the models used do not consistently account for confounders. While our study aligns with the core MR assumptions and includes pleiotropy analysis to reduce confounding, we cannot fully exclude its impact on the causal link between molecular traits and HF. The study’s sample sizes for mQTL, GWAS data, and genetic variants tied to protein expression are limited, which may cause some HF-related genes to be missed. Additionally, the scarcity of GWAS datasets, especially those on cellular aging, restricts the bidirectional MR analysis of causality. Even with sensitivity analyses, pleiotropy assessment is still not precise, necessitating more individual-level data for refined stratified analysis. The study is based mainly on European population data, and while it was tested in East Asian groups, the specificity of genetic mutations restricts the wider applicability of the findings. Further functional experiments are required to validate these conclusions.

## Conclusion

This study has increased our understanding of how senescence may influence the biological mechanisms of HF. It has shown that methylation and gene expression related to *CDKN1A* may play a role in initiating senescence in HF cardiomyocytes. It may help identify potential new therapeutic targets for HF and advance fundamental research on the role of cellular senescence in the disease.

## Data Availability

The datasets presented in this study can be found in online repositories. The names of the repository/repositories and accession number(s) can be found in the article/Supplementary Material.

## References

[B1] Alcendor R. R. Gao S. Zhai P. Zablocki D. Holle E. Yu X. (2007). Sirt1 regulates aging and resistance to oxidative stress in the heart. Circ. Res. 100 (10), 1512–1521. 10.1161/01.RES.0000267723.65696.4a 17446436

[B2] Avelar R. A. Ortega J. G. Tacutu R. Tyler E. J. Bennett D. Binetti P. (2020). A multidimensional systems biology analysis of cellular senescence in aging and disease. Genome Biol. 21 (1), 91. 10.1186/s13059-020-01990-9 32264951 PMC7333371

[B3] Baman J. R. Ahmad F. S. (2020). Heart failure. Jama 324 (10), 1015. 10.1001/jama.2020.13310 32749448

[B4] Benjamini Y. Hochberg Y. (2018). Controlling the false discovery rate: a practical and powerful approach to multiple testing. J. R. Stat. Soc. Ser. B Methodol. 57 (1), 289–300. 10.1111/j.2517-6161.1995.tb02031.x

[B5] Bloom S. I. Islam M. T. Lesniewski L. A. Donato A. J. (2023). Mechanisms and consequences of endothelial cell senescence. Nat. Rev. Cardiol. 20 (1), 38–51. 10.1038/s41569-022-00739-0 35853997 PMC10026597

[B6] Bowden J. Davey Smith G. Burgess S. (2015). Mendelian randomization with invalid instruments: effect estimation and bias detection through Egger regression. Int. J. Epidemiol. 44 (2), 512–525. 10.1093/ije/dyv080 26050253 PMC4469799

[B7] Burgess S. Dudbridge F. Thompson S. G. (2016). Combining information on multiple instrumental variables in Mendelian randomization: comparison of allele score and summarized data methods. Stat. Med. 35 (11), 1880–1906. 10.1002/sim.6835 26661904 PMC4832315

[B8] Cao E. Y. Ouyang J. F. Rackham O. J. L. (2020). GeneSwitches: ordering gene expression and functional events in single-cell experiments. Bioinformatics 36 (10), 3273–3275. 10.1093/bioinformatics/btaa099 32058565

[B9] Carvalho C. L'Hôte V. Courbeyrette R. Kratassiouk G. Pinna G. Cintrat J. C. (2019). Glucocorticoids delay RAF-induced senescence promoted by EGR1. J. Cell. Sci. 132 (16), jcs230748. 10.1242/jcs.230748 31371485

[B10] Cazzalini O. Scovassi A. I. Savio M. Stivala L. A. Prosperi E. (2010). Multiple roles of the cell cycle inhibitor p21(CDKN1A) in the DNA damage response. Mutat. Res. 704 (1-3), 12–20. 10.1016/j.mrrev.2010.01.009 20096807

[B11] Chatsirisupachai K. Palmer D. Ferreira S. de Magalhães J. P. (2019). A human tissue-specific transcriptomic analysis reveals a complex relationship between aging, cancer, and cellular senescence. Aging Cell. 18 (6), e13041. 10.1111/acel.13041 31560156 PMC6826163

[B12] Chen J. Chapski D. J. Jong J. Awada J. Wang Y. Slamon D. J. (2023). Integrative transcriptomics and cell systems analyses reveal protective pathways controlled by Igfbp-3 in anthracycline-induced cardiotoxicity. Faseb J. 37 (6), e22977. 10.1096/fj.202201885RR 37219486 PMC10286824

[B13] Davey Smith G. Hemani G. (2014). Mendelian randomization: genetic anchors for causal inference in epidemiological studies. Hum. Mol. Genet. 23 (R1), R89–R98. 10.1093/hmg/ddu328 25064373 PMC4170722

[B14] Dobbyn A. Huckins L. M. Boocock J. Sloofman L. G. Glicksberg B. S. Giambartolomei C. (2018). Landscape of conditional eQTL in dorsolateral prefrontal cortex and Co-localization with schizophrenia GWAS. Am. J. Hum. Genet. 102 (6), 1169–1184. 10.1016/j.ajhg.2018.04.011 29805045 PMC5993513

[B15] Evangelou K. Vasileiou P. V. S. Papaspyropoulos A. Hazapis O. Petty R. Demaria M. (2023). Cellular senescence and cardiovascular diseases: moving to the “heart” of the problem. Physiol. Rev. 103 (1), 609–647. 10.1152/physrev.00007.2022 36049114

[B16] Freshour S. L. Kiwala S. Cotto K. C. Coffman A. C. McMichael J. F. Song J. J. (2021). Integration of the drug-gene interaction database (DGIdb 4.0) with open crowdsource efforts. Nucleic Acids Res. 49 (D1), D1144–d1151. 10.1093/nar/gkaa1084 33237278 PMC7778926

[B17] Gombos T. Förhécz Z. Pozsonyi Z. Jánoskuti L. Prohászka Z. (2008). Interaction of serum 70-kDa heat shock protein levels and HspA1B (+1267) gene polymorphism with disease severity in patients with chronic heart failure. Cell. Stress Chaperones 13 (2), 199–206. 10.1007/s12192-007-0001-5 18759004 PMC2673893

[B18] Hernandez-Segura A. Nehme J. Demaria M. (2018). Hallmarks of cellular senescence. Trends Cell. Biol. 28 (6), 436–453. 10.1016/j.tcb.2018.02.001 29477613

[B19] Hu C. Zhang X. Teng T. Ma Z. G. Tang Q. Z. (2022). Cellular senescence in cardiovascular diseases: a systematic review. Aging Dis. 13 (1), 103–128. 10.14336/ad.2021.0927 35111365 PMC8782554

[B20] Huang S. Xu M. Liu L. Yang J. Wang H. Wan C. (2020). Autophagy is involved in the protective effect of p21 on LPS-induced cardiac dysfunction. Cell. Death Dis. 11 (7), 554. 10.1038/s41419-020-02765-7 32694519 PMC7374585

[B21] Kamat M. A. Blackshaw J. A. Young R. Surendran P. Burgess S. Danesh J. (2019). PhenoScanner V2: an expanded tool for searching human genotype-phenotype associations. Bioinformatics 35 (22), 4851–4853. 10.1093/bioinformatics/btz469 31233103 PMC6853652

[B22] Karimian A. Ahmadi Y. Yousefi B. (2016). Multiple functions of p21 in cell cycle, apoptosis and transcriptional regulation after DNA damage. DNA Repair (Amst) 42, 63–71. 10.1016/j.dnarep.2016.04.008 27156098

[B23] Katoh M. Nomura S. Yamada S. Ito M. Hayashi H. Katagiri M. (2024). Vaccine therapy for heart failure targeting the inflammatory cytokine Igfbp7. Circulation 150 (5), 374–389. 10.1161/circulationaha.123.064719 38991046

[B31] Kurki M. I. Karjalainen J. Palta P. Sipilä T. P. Kristiansson K. Donner K. M. (2023). FinnGen provides genetic insights from a well-phenotyped isolated population. Nature 613 (7944), 508–518. 10.1038/s41586-022-05473-8 36653562 PMC9849126

[B24] Lee B. Yao X. Shen L. Alzheimer’s Disease Neuroimaging Initiative (2022). Integrative analysis of summary data from GWAS and eQTL studies implicates genes differentially expressed in Alzheimer's disease. BMC Genomics 23 (Suppl. 4), 414. 10.1186/s12864-022-08584-8 35655140 PMC9161451

[B25] Li H. Hastings M. H. Rhee J. Trager L. E. Roh J. D. Rosenzweig A. (2020). Targeting age-related pathways in heart failure. Circ. Res. 126 (4), 533–551. 10.1161/circresaha.119.315889 32078451 PMC7041880

[B26] Liu K. Chen S. Lu R. (2021). Identification of important genes related to ferroptosis and hypoxia in acute myocardial infarction based on WGCNA. Bioengineered 12 (1), 7950–7963. 10.1080/21655979.2021.1984004 34565282 PMC8806940

[B27] López-Domínguez J. A. Rodríguez-López S. Ahumada-Castro U. Desprez P. Y. Konovalenko M. Laberge R. M. (2021). Cdkn1a transcript variant 2 is a marker of aging and cellular senescence. Aging (Albany NY) 13 (10), 13380–13392. 10.18632/aging.203110 34035185 PMC8202863

[B28] López-Otín C. Blasco M. A. Partridge L. Serrano M. Kroemer G. (2013). The hallmarks of aging. Cell. 153 (6), 1194–1217. 10.1016/j.cell.2013.05.039 23746838 PMC3836174

[B29] Lu A. T. Hannon E. Levine M. E. Crimmins E. M. Lunnon K. Mill J. (2017). Genetic architecture of epigenetic and neuronal ageing rates in human brain regions. Nat. Commun. 8, 15353. 10.1038/ncomms15353 28516910 PMC5454371

[B30] Luo X. Yin J. Dwyer D. Yamawaki T. Zhou H. Ge H. (2021). Chamber-enriched gene expression profiles in failing human hearts with reduced ejection fraction. Sci. Rep. 11 (1), 11839. 10.1038/s41598-021-91214-2 34088950 PMC8178406

[B32] North B. J. Sinclair D. A. (2012). The intersection between aging and cardiovascular disease. Circ. Res. 110 (8), 1097–1108. 10.1161/circresaha.111.246876 22499900 PMC3366686

[B33] Salih A. M. Pujadas E. R. Campello V. M. McCracken C. Harvey N. C. Neubauer S. (2023). Image-based biological heart age estimation reveals differential aging patterns across cardiac chambers. J. Magn. Reson Imaging 58 (6), 1797–1812. 10.1002/jmri.28675 36929232 PMC10947470

[B34] Saul D. Kosinsky R. L. Atkinson E. J. Doolittle M. L. Zhang X. LeBrasseur N. K. (2022). A new gene set identifies senescent cells and predicts senescence-associated pathways across tissues. Nat. Commun. 13 (1), 4827. 10.1038/s41467-022-32552-1 35974106 PMC9381717

[B35] Shah S. Henry A. Roselli C. Lin H. Sveinbjörnsson G. Fatemifar G. (2020). Genome-wide association and Mendelian randomisation analysis provide insights into the pathogenesis of heart failure. Nat. Commun. 11 (1), 163. 10.1038/s41467-019-13690-5 31919418 PMC6952380

[B36] Shahim B. Kapelios C. J. Savarese G. Lund L. H. (2023). Global public health burden of heart failure: an updated review. Card. Fail Rev. 9, e11. 10.15420/cfr.2023.05 37547123 PMC10398425

[B37] Skrivankova V. W. Richmond R. C. Woolf B. A. R. Davies N. M. Swanson S. A. VanderWeele T. J. (2021). Strengthening the reporting of observational studies in epidemiology using mendelian randomisation (STROBE-MR): explanation and elaboration. Bmj 375, n2233. 10.1136/bmj.n2233 34702754 PMC8546498

[B38] Steenman M. Lande G. (2017). Cardiac aging and heart disease in humans. Biophys. Rev. 9 (2), 131–137. 10.1007/s12551-017-0255-9 28510085 PMC5418492

[B39] Steinberg J. Southam L. Roumeliotis T. I. Clark M. J. Jayasuriya R. L. Swift D. (2021). A molecular quantitative trait locus map for osteoarthritis. Nat. Commun. 12 (1), 1309. 10.1038/s41467-021-21593-7 33637762 PMC7910531

[B40] Stuart T. Butler A. Hoffman P. Hafemeister C. Papalexi E. Mauck W. M. (2019). Comprehensive integration of single-cell data. Cell. 177 (7), 1888–1902. 10.1016/j.cell.2019.05.031 31178118 PMC6687398

[B41] Sun B. B. Chiou J. Traylor M. Benner C. Hsu Y. H. Richardson T. G. (2023). Plasma proteomic associations with genetics and health in the UK Biobank. Nature 622 (7982), 329–338. 10.1038/s41586-023-06592-6 37794186 PMC10567551

[B42] Sweet M. E. Cocciolo A. Slavov D. Jones K. L. Sweet J. R. Graw S. L. (2018). Transcriptome analysis of human heart failure reveals dysregulated cell adhesion in dilated cardiomyopathy and activated immune pathways in ischemic heart failure. BMC Genomics 19 (1), 812. 10.1186/s12864-018-5213-9 30419824 PMC6233272

[B43] Tao H. Xu W. Qu W. Gao H. Zhang J. Cheng X. (2021). Loss of ten-eleven translocation 2 induces cardiac hypertrophy and fibrosis through modulating ERK signaling pathway. Hum. Mol. Genet. 30 (10), 865–879. 10.1093/hmg/ddab046 33791790

[B44] Teumer A. (2018). Common methods for performing mendelian randomization. Front. Cardiovasc Med. 5, 51. 10.3389/fcvm.2018.00051 29892602 PMC5985452

[B45] Võsa U. Claringbould A. Westra H. J. Bonder M. J. Deelen P. Zeng B. (2021). Large-scale cis- and trans-eQTL analyses identify thousands of genetic loci and polygenic scores that regulate blood gene expression. Nat. Genet. 53 (9), 1300–1310. 10.1038/s41588-021-00913-z 34475573 PMC8432599

[B46] Wang C. Taskinen J. H. Segersvärd H. Immonen K. Kosonen R. Tolva J. M. (2022). Alterations of cardiac protein kinases in cyclic nucleotide-dependent signaling pathways in human ischemic heart failure. Front. Cardiovasc Med. 9, 919355. 10.3389/fcvm.2022.919355 35783854 PMC9247256

[B47] Wang L. Yu P. Zhou B. Song J. Li Z. Zhang M. (2020). Single-cell reconstruction of the adult human heart during heart failure and recovery reveals the cellular landscape underlying cardiac function. Nat. Cell. Biol. 22 (1), 108–119. 10.1038/s41556-019-0446-7 31915373

[B48] Wu Y. Yang S. Ma J. Chen Z. Song G. Rao D. (2022). Spatiotemporal immune landscape of colorectal cancer liver metastasis at single-cell level. Cancer Discov. 12 (1), 134–153. 10.1158/2159-8290.Cd-21-0316 34417225

[B49] Wu Y. Zeng J. Zhang F. Zhu Z. Qi T. Zheng Z. (2018). Integrative analysis of omics summary data reveals putative mechanisms underlying complex traits. Nat. Commun. 9 (1), 918. 10.1038/s41467-018-03371-0 29500431 PMC5834629

[B50] Xie S. Xu S. C. Deng W. Tang Q. (2023). Metabolic landscape in cardiac aging: insights into molecular biology and therapeutic implications. Signal Transduct. Target Ther. 8 (1), 114. 10.1038/s41392-023-01378-8 36918543 PMC10015017

[B51] Yin K. Patten D. Gough S. de Barros Gonçalves S. Chan A. Olan I. (2022). Senescence-induced endothelial phenotypes underpin immune-mediated senescence surveillance. Genes. Dev. 36 (9-10), 533–549. 10.1101/gad.349585.122 35618311 PMC9186388

[B52] Zhang S. Song Z. An L. Liu X. Hu X. W. Naz A. (2019). WD40 repeat and FYVE domain containing 3 is essential for cardiac development. Cardiovasc Res. 115 (8), 1320–1331. 10.1093/cvr/cvy285 30428088

[B53] Zhang Y. Li D. Zhu Z. Chen S. Lu M. Cao P. (2022). Evaluating the impact of metformin targets on the risk of osteoarthritis: a mendelian randomization study. Osteoarthr. Cartil. 30 (11), 1506–1514. 10.1016/j.joca.2022.06.010 35803489

[B54] Zheng J. Ma Y. Guo X. Wu J. (2023). Immunological characterization of stroke-heart syndrome and identification of inflammatory therapeutic targets. Front. Immunol. 14, 1227104. 10.3389/fimmu.2023.1227104 37965346 PMC10642553

[B55] Zhou D. Y. Su X. Wu Y. Yang Y. Zhang L. Cheng S. (2024). Decreased CNNM2 expression in prefrontal cortex affects sensorimotor gating function, cognition, dendritic spine morphogenesis and risk of schizophrenia. Neuropsychopharmacology 49 (2), 433–442. 10.1038/s41386-023-01732-y 37715107 PMC10724213

[B56] Zhu Z. Zhang F. Hu H. Bakshi A. Robinson M. R. Powell J. E. (2016). Integration of summary data from GWAS and eQTL studies predicts complex trait gene targets. Nat. Genet. 48 (5), 481–487. 10.1038/ng.3538 27019110

[B57] Zou M. Liang Q. Zhang W. Zhu Y. Xu Y. (2023). Endoplasmic reticulum stress related genome-wide Mendelian randomization identifies therapeutic genes for ulcerative colitis and Crohn's disease. Front. Genet. 14, 1270085. 10.3389/fgene.2023.1270085 37860672 PMC10583552

