# Supplementary material for: CDKN1A as a target of senescence in heart failure: insights from a multiomics study
Source: Front Pharmacol. 2024 Oct 23;15:1446300. doi: 10.3389/fphar.2024.1446300 (PMC11541717; doi:10.3389/fphar.2024.1446300)
Supplement: Supplementary file 2 [file DataSheet1.docx]

***Additional file 2***

**CDKN1A as a target of senescence in heart failure: insights from a multiomics study**

Rutao Bian^#1,2^, Li Zhang^#1,2^, Dongyu Li^1,2^, Xuegong Xu^1,2^

**Authors and Affiliations**

1, Department of Cardiology, Zhengzhou Hospital of Traditional Chinese Medicine, Zhengzhou, Henan, China

Rutao Bian, Li Zhang, Dongyu Li & Xuegong Xu

2, The Affiliated Zhengzhou Hospital of Traditional Chinese Medicine, Henan University of Chinese Medicine, Zhengzhou, Henan, China

Rutao Bian, Li Zhang, Dongyu Li & Xuegong Xu

#Contributed equally

**STROBE-MR checklist of recommended items to address in reports of Mendelian randomization studies**^1^ ^2^

| **Item No.** | **Section** | **Checklist item** |  | **Relevant text from manuscript** |
| --- | --- | --- | --- | --- |
| 1 | **TITLE and ABSTRACT** | Indicate Mendelian randomization (MR) as the study’s design in the title and/or the abstract if that is a main purpose of the study |  | Title: CDKN1A as a target of senescence in heart failure: insights from a multiomics study  Abstract: Background  Cardiomyocyte senescence plays a crucial role as a pathological mechanism in heart failure (HF). However, the exact triggering factors and underlying causes of HF onset and progression are still not fully understood.  Objectives  By integrating multi-omics data, this study aimed to determine the genetic associations between cardiomyocyte and HF using cell senescence-related genes (SRGs).  Methods  The study utilized the CellAge database and the SenMayo dataset, combined with high-resolution single-cell RNA sequencing (scRNA-seq) data, to identify SRG and examine differences in cardiac cell expression. To explore the causal relationship with HF using Mendelian Randomization (MR). Genetic variations influencing gene expression, DNA methylation, and protein expression (cis-eQTL, cis-mQTL, and cis-pQTL) were analyzed using the two-sample MR (TSMR) and summary-data-based MR (SMR). Additionally, Bayesian colocalization analysis, germline genetic variation, and bulk RNA data were employed to strengthen the reliability of the results. The application potential of therapeutic targets is ultimately assessed by evaluating their druggability.  Results  The expression of 39 SRGs in cardiomyocytes was identified. In the discovery set revealed that CDKN1A (OR = 1.09, 95% confidence interval (CI) 1.02–1.15, FDR = 0.048) could be causally related to HF, and the results are also replicated in the validation set (OR = 1.20, 95% confidence interval (CI) 1.10–1.30, FDR < 0.0001). Based on the SMR method, CDKN1A was confirmed as a candidate pathogenic gene for HF, and its methylation (cg03714916, cg08179530) was associated with HF risk loci. The result is validated by Bayesian colocalization analysis, genetic variations, and bulk RNA data. The druggability analysis identified two potential therapeutic drugs.  Conclusion  Based on multi-omics data, this study uncovered the reciprocal regulation of cardiomyocyte senescence through CDKN1A, providing potential targets for HF drug development. |
|  | **INTRODUCTION** |  |  |  |
| 2 | **Background** | Explain the scientific background and rationale for the reported study. What is the exposure? Is a potential causal relationship between exposure and outcome plausible? Justify why MR is a helpful method to address the study question |  | Heart failure (HF) is a complex syndrome characterized by decreased filling or poor ejection, along with symptoms such as dyspnea and fatigue [1]. The role of aging as a major risk factor for cardiovascular disease is often overlooked [2]. Even in the absence of systemic risk factors like smoking, dyslipidemia, hypertension, and diabetes, intrinsic cardiac aging can lead to a decline in cardiac structure and function in the elderly. Epigenetic changes are implicated in various age-related cardiac diseases, such as ischemic heart disease, which might make the heart more susceptible to aging. The accelerated aging of the population increases the risk of HF, particularly in individuals with chronic diseases, which imposes a significant economic burden on the public health system [3]. Therefore, understanding the triggers of cardiac aging and identifying key molecular targets that contribute to it is essential.  Several mechanisms contribute to senescence, including increased oxidative stress, stem cell depletion, altered cell communication, reduced genomic stability, shortening of telomeres, epigenetic changes, disrupted protein homeostasis, impaired nutrient metabolism, and mitochondrial dysfunction, which lead to changes at the molecular, cellular, tissue, and organ levels[4,5]. Consequently, hypertrophy, fibrosis, protein misfolding, mitochondrial dysfunction, and an increase in sympathetic nerve activity are observed [6,7]. Senescence-related genes (SRGs) in human tissues are linked to accelerated aging. Furthermore, these SRGs are not restricted to specific tissues, indicating that their expression varies between different organs and cell types, which results in distinct biological functions [8]. Cardiomyocytes, which make up 30% to 40% of the total cardiac cells, undergo morphological and functional changes with age. Studies have show that elderly hearts contain a significant number of senescent cells, which may be responsible for structural and functional changes as a result of hypertrophy of the left ventricle, a decrease in diastolic function, and pathological changes including myocardial fibrosis, extracellular matrix remodeling, and conduction block [9].  Meanwhile, some studies have partially revealed the connection between aging-related gene regulation and HF. Currently, traditional observational studies have identified associations between specific cell senescence-related genes and HF risk. For example, mild to moderate expression of Sirt1 can delay cardiac aging [10], and vaccine therapies targeting Igfbp7 may help prevent the development of HF [11,12]. However, the underlying mechanisms involving epigenetic changes remain unclear. Genome-wide association studies (GWAS) have uncovered intricate details of the pathophysiology of complex diseases, providing important insights. These extensive studies have identified numerous disease-related genetic loci, further enhancing our understanding of the critical role genetic factors play in disease etiology. |
| 3 | **Objectives** | State specific objectives clearly, including pre-specified causal hypotheses (if any). State that MR is a method that, under specific assumptions, intends to estimate causal effects |  | Multi-omics data combined with Mendelian randomization analysis can reveal new insights about disease pathogenesis and therapeutic targets. This study uses ScRNA-seq, bulk RNA, and Mendelian randomization (MR) to identify risk loci for HF cardiomyocyte senescence, providing a scientific rationale to improve the prevention and treatment of HF. |
|  | **METHODS** |  |  |  |
| 4 | **Study design and data sources** | Present key elements of the study design early in the article. Consider including a table listing sources of data for all phases of the study. For each data source contributing to the analysis, describe the following: |  |  |
|  | a) | Setting: Describe the study design and the underlying population, if possible. Describe the setting, locations, and relevant dates, including periods of recruitment, exposure, follow-up, and data collection, when available. |  | An overview of the study's workflow can be found in Figure 1. We obtained 307 genes from the CellAge database [19,20] and 125 genes from the SenMayo set defined by Saul et al [21]. With duplicate genes removed, 413 senescence-related genes were listed (Additional file 1: Supplementary Table S1). Initially, differential gene analysis in cardiomyocytes was performed using ScRNA-seq dataset. two-sample MR (TSMR) and Summary data-based Mendelian randomization (SMR) [14] analysis were used to identify potential risk loci for HF using the cardiomyocyte SRGs. In addition, bulk RNA-seq of various types of HF was performed to assess the reliability of the findings. This study followed the STROBE-MR[22] (Strengthening the reporting of observational studies in epidemiology using mendelian randomization) guidelines and the STROBE-MR reporting checklist (Additional file 2: STROBE-MR checklist). |
|  | b) | Participants: Give the eligibility criteria, and the sources and methods of selection of participants. Report the sample size, and whether any power or sample size calculations were carried out prior to the main analysis |  | A detailed description of the data sources is shown in Additional file 1：Supplementary Table S2 |
|  | c) | Describe measurement, quality control and selection of genetic variants |  | IVs related to single nucleotide polymorphisms (SNPs) of the target gene were extracted from the dataset. The MR analysis aimed to investigate the causal relationship between DEGs and the risk of HF, utilizing these SNPs as IVs. SNPs with significance threshold < 5.0E-08, minor allele frequency (MAF) > 0.01, SNP allele frequency difference < 0.2, and maximum allele frequency difference ratio < 0.05, were identified. Only robust IVs with F-statistics exceeding 10 were retained. |
|  | d) | For each exposure, outcome, and other relevant variables, describe methods of assessment and diagnostic criteria for diseases |  | “A detailed description of the data sources is shown in Additional file 1：Supplementary Table S2.” |
|  | e) | Provide details of ethics committee approval and participant informed consent, if relevant |  | NA |
| 5 | **Assumptions** | Explicitly state the three core IV assumptions for the main analysis (relevance, independence and exclusion restriction) as well assumptions for any additional or sensitivity analysis |  | To validate the IVs in blood data, three key assumptions were imposed: (1) a strong correlation between genetic instruments and the exposure, (2) no correlation with potential confounders, and (3) no correlation with confounders influencing the exposure-outcome relationship. |
| 6 | **Statistical methods: main analysis** | Describe statistical methods and statistics used |  |  |
|  | a) | Describe how quantitative variables were handled in the analyses (i.e., scale, units, model) |  | To calculate MR estimates for individual SNPs, we used the Wald ratio method [40], the inverse-variance weighted (IVW) model [41], which utilizes multiple IVs for genes. To minimize potential omissions of target genes, FDR < 0.05 was established [42]. MR-Egger [43] intercepts and Cochran's Q were used to measure horizontal pleiotropy and heterogeneity. PhenoScanner [44] was also used to explore associations between identified QTLs and other traits. The leave-one-out analysis was utilized to identify potential outliers that could introduce significant bias into the results. Subsequently, these outliers were excluded, and the MR analyses were re-performed. |
|  | b) | Describe how genetic variants were handled in the analyses and, if applicable, how their weights were selected |  | IVs related to single nucleotide polymorphisms (SNPs) of the target gene were extracted from the dataset. The MR analysis aimed to investigate the causal relationship between DEGs and the risk of HF, utilizing these SNPs as IVs. SNPs with significance threshold < 5.0E-08, minor allele frequency (MAF) > 0.01, SNP allele frequency difference < 0.2, and maximum allele frequency difference ratio < 0.05, were identified. Only robust IVs with F-statistics exceeding 10 were retained. To validate the IVs in blood data, three key assumptions were imposed: (1) a strong correlation between genetic instruments and the exposure, (2) no correlation with potential confounders, and (3) no correlation with confounders influencing the exposure-outcome relationship. |
|  | c) | Describe the MR estimator (e.g. two-stage least squares, Wald ratio) and related statistics. Detail the included covariates and, in case of two-sample MR, whether the same covariate set was used for adjustment in the two samples |  | To calculate MR estimates for individual SNPs, we used the Wald ratio method [40], the inverse-variance weighted (IVW) model [41], which utilizes multiple IVs for genes. To minimize potential omissions of target genes, FDR < 0.05 was established [42]. MR-Egger [43] intercepts and Cochran's Q were used to measure horizontal pleiotropy and heterogeneity. PhenoScanner [44] was also used to explore associations between identified QTLs and other traits. The leave-one-out analysis was utilized to identify potential outliers that could introduce significant bias into the results. Subsequently, these outliers were excluded, and the MR analyses were re-performed. |
|  | d) | Explain how missing data were addressed | NA | NA |
|  | e) | If applicable, indicate how multiple testing was addressed |  | ….To minimize potential omissions of target genes, FDR < 0.05 was established [33]……  To establish the final causal relationship, the following criteria must be met in the three-step SMR [37]: (1) FDR < 0.05, (2) HEIDI >0.01, and (3) eQTL and mQTL should correspond to the same gene symbol.  Bayesian colocalization analyses test whether GWAS summary data and eQTL share causal variants. It assesses the posterior probability of each hypothesis based on five hypotheses (H0, no association with either GWAS or QTL at the locus; H1, the association only with GWAS; H2, the association only with QTL; H3, the association only with GWAS and QTL but not colocalized; H4, colocalization of GWAS and QTL). A colocalization analysis was performed on all SNPs within 100 kb of the top SNP of the probe. Several loci with PPH4 ≥ 0.5 appear to align with the colocalization suggested by PPH4 ≥ 0.8 [38]. |
| 7 | **Assessment of assumptions** | Describe any methods or prior knowledge used to assess the assumptions or justify their validity |  |  |
| 8 | **Sensitivity analyses and additional analyses** | Describe any sensitivity analyses or additional analyses performed (e.g. comparison of effect estimates from different approaches, independent replication, bias analytic techniques, validation of instruments, simulations) |  | MR-Egger [43] intercepts and Cochran's Q were used to measure horizontal pleiotropy and heterogeneity. PhenoScanner [44] was also used to explore associations between identified QTLs and other traits. The leave-one-out analysis was utilized to identify potential outliers that could introduce significant bias into the results. Subsequently, these outliers were excluded, and the MR analyses were re-performed. |
| 9 | **Software and pre-registration** |  |  |  |
|  | a) | Name statistical software and package(s), including version and settings used |  | The SMR analysis and HEIDI testing were conducted using version 1.3.1 of the SMR software available at https://yanglab.westlake.edu.cn/software/smr/#Download. Two-sample MR analysis was performed utilizing the "TwoSampleMR (version 4.2.2)" package within R software (version 0.5.6). Colocalization analysis was carried out using the "coloc (version 3.3.0)" R package. Differential analysis of datasets utilized the "Limma (version 1.2.6)" package, while result visualization was achieved using the "forestploter (version 3.0.1)" package. |
|  | b) | State whether the study protocol and details were pre-registered (as well as when and where) | NA |  |
|  | **RESULTS** |  |  |  |
| 10 | **Descriptive data** |  |  |  |
|  | a) | Report the numbers of individuals at each stage of included studies and reasons for exclusion. Consider use of a flow diagram |  | Figure 1. Flowchart of the analyses performed. |
|  | b) | Report summary statistics for phenotypic exposure(s), outcome(s), and other relevant variables (e.g. means, SDs, proportions) | NA | NA |
|  | c) | If the data sources include meta-analyses of previous studies, provide the assessments of heterogeneity across these studies | NA | NA |
|  | d) | For two-sample MR:  i.  Provide justification of the similarity of the genetic variant-exposure associations between the exposure and outcome samples  ii.  Provide information on the number of individuals who overlap between the exposure and outcome studies |  | The study data predominantly comprise individuals of European ancestry. |
| 11 | **Main results** |  |  |  |
|  | a) | Report the associations between genetic variant and exposure, and between genetic variant and outcome, preferably on an interpretable scale |  | After eliminating outliers for the multiple regression analysis, the F statistics of all SNPs surpassed 10, suggesting that these SNPs are appropriate for utilization as robust instrumental factors. The findings suggest that heat shock protein family A member 1B (HSPA1B), cyclin-dependent kinase inhibitor 1A (CDKN1A) may serve as potential genes, and the overall causal effects of cis-eQTL of all genes on HF were summarized (Figure 3, Additional file 1: Supplementary Table S5). The MR-Egger method was utilized to assess the sensitivity and directional pleiotropy of different genes, and the Egger intercept results of MR-Egger indicated that all variable P > 0.05, suggesting no significant pleiotropy was present (Additional file 1: Supplementary Table S7). Additionally, the leave-one-out test results demonstrated that, upon removing each SNP in turn, the remaining SNPs yielded similar analysis results to the inclusion of all SNPs, with no SNP significantly influencing the estimated causal values. Furthermore, the FinnGen study validated CDKN1A as a potential risk locus for HF (Figure 3, Additional file 1: Supplementary Table S6). |
|  | b) | Report MR estimates of the relationship between exposure and outcome, and the measures of uncertainty from the MR analysis, on an interpretable scale, such as odds ratio or relative risk per SD difference |  | Additional file 1: Supplementary Table S5, S6, S7. |
|  | c) | If relevant, consider translating estimates of relative risk into absolute risk for a meaningful time period | NA |  |
|  | d) | Consider plots to visualize results (e.g. forest plot, scatterplot of associations between genetic variants and outcome versus between genetic variants and exposure) |  | Figure 3. Cardiomyocyte senescence and HF risk estimation by TSMR analysis. |
| 12 | **Assessment of assumptions** |  |  |  |
|  | a) | Report the assessment of the validity of the assumptions |  | After eliminating outliers for the multiple regression analysis, the F statistics of all SNPs surpassed 10, suggesting that these SNPs are appropriate for utilization as robust instrumental factors. The findings suggest that heat shock protein family A member 1B (HSPA1B), cyclin-dependent kinase inhibitor 1A (CDKN1A) may serve as potential genes, and the overall causal effects of cis-eQTL of all genes on HF were summarized (Figure 3, Additional file 1: Supplementary Table S5). |
|  | b) | Report any additional statistics (e.g., assessments of heterogeneity across genetic variants, such as *I^2^*, Q statistic or E-value) |  | The MR-Egger method was utilized to assess the sensitivity and directional pleiotropy of different genes, and the Egger intercept results of MR-Egger indicated that all variable P > 0.05, suggesting no significant pleiotropy was present (Additional file 1: Supplementary Table S7). Additionally, the leave-one-out test results demonstrated that, upon removing each SNP in turn, the remaining SNPs yielded similar analysis results to the inclusion of all SNPs, with no SNP significantly influencing the estimated causal values. Furthermore, the FinnGen study validated CDKN1A as a potential risk locus for HF (Figure 3, Additional file 1: Supplementary Table S6). |
| 13 | **Sensitivity analyses and additional analyses** |  |  |  |
|  | a) | Report any sensitivity analyses to assess the robustness of the main results to violations of the assumptions |  | The MR-Egger method was utilized to assess the sensitivity and directional pleiotropy of different genes, and the Egger intercept results of MR-Egger indicated that all variable P > 0.05, suggesting no significant pleiotropy was present (Additional file 1: Supplementary Table S7). Additionally, the leave-one-out test results demonstrated that, upon removing each SNP in turn, the remaining SNPs yielded similar analysis results to the inclusion of all SNPs, with no SNP significantly influencing the estimated causal values. Furthermore, the FinnGen study validated CDKN1A as a potential risk locus for HF (Figure 3, Additional file 1: Supplementary Table S6). |
|  | b) | Report results from other sensitivity analyses or additional analyses |  | SMR analysis of senescence genome-wide cis-eQTLs and HF  The SMR results of 2055 SNPs representing SRG expressions were associated with the experience of HF (Additional file 1: Supplementary Table S8). As a result of test correction, only CDKN1A showed a strong association (Figure 4, FDR = 2.07E-05, Additional file 1: Supplementary Table S8), whereas the subsequent HEIDI test excluded pleiotropy. Additionally, the CDKN1A and HF have been linked in the FinnGen cohort (Additional file 1: Supplementary Table S8, FDR = 1.20E-09). The findings of our study suggest an increased risk of HF in people with high CDKN1A expression.  SMR analysis of SGRs genome-wide cis-mQTLs and HF  The causal relationship between DNAm in SRGs and HF was examined. According to SMR analysis of mQTL data, 195 CpG sites were identified (Figure 5A，Additional file 1: Supplementary Table S9). These sites corresponded to nine genes associated with HF. According to the HEIDI test, CDKN1A exhibited six independent sites, while EGR1, ADCY5, and MAP3K5 each exhibited one. In the FinnGen cohort, only cg03714916 (FDR = 3.54E-06) and cg08179530 (FDR = 5.96E-05) passed validation (Additional file 1: Supplementary Table S9). There was a positive correlation between CDKN1A expression and HF onset at site cg03714916 (Figure 5B). In contrast, a negative correlation was found between CDKN1A expression and HF onset at site cg08179530 (Figure 5B), indicating an association between higher CDKN1A expression and HF onset. Therefore, lower levels of DNA methylation in the CDKN1A enhancer region may stimulate gene expression, thereby increasing the risk of HF.  As a result of investigating the relationship between mQTL and eQTL, we observed significant interactions between 59 sites of 21 genes (Additional file 1: Supplementary Table S10). Our analysis of the results of the previous two steps found a significant association between cg08179530 and a reduced risk of HF (PSMR = 1.47E-08) and a significant association between cg15474579 and an increased risk of HF (PSMR = 5.48E-36). A three-step SMR analysis showed that the SNP signals and CDKN1A in the HF GWAS, mQTL, and eQTL data studies were highly significant.  Sensitivity analysis  A colocalization analysis was conducted to assess the influence of linkage disequilibrium. A posterior probability (PP.H4) of shared causality between trans-gene expression and HF greater than 0.50 suggests that HF GWAS and eQTL are colocalized. Bayesian colocalization results indicate that CDKN1A and HF share genetic variation in the Consortium dataset (Figure 6A, Additional file 1: Supplementary Table S11). However, CDKN1A and HF shared no genetic variation in the FinnGen cohort (Figure 6B, Additional file 1: Supplementary Table S11). In addition, phenotype scanning has shown that CDKN1A is associated with trunk fat-free mass, trunk predicted mass, and triglycerides, while total cholesterol is associated with atopic dermatitis ( Additional file 1: Supplementary Table S12). |
|  | c) | Report any assessment of direction of causal relationship (e.g., bidirectional MR) | NA |  |
|  | d) | When relevant, report and compare with estimates from non-MR analyses |  | Figure 4, Figure 5, Figure 6. |
|  | e) | Consider additional plots to visualize results (e.g., leave-one-out analyses) |  |  |
|  | **DISCUSSION** |  |  |  |
| 14 | **Key results** | Summarize key results with reference to study objectives |  | The TSMR analysis initially suggested HSPA1B, CDKN1A as potential risk loci. However, the FinnGen study confirmed only CDKN1A as a potential risk locus for HF. To further validate these findings, Bayesian colocalization analysis, genetic variation in the germline, and extensive RNA data were utilized. The study also investigated the connections between specific SNPs and CpG sites and the occurrence of HF, as well as the relationship between cellular characteristics and CDKN1A expression. Strong evidence indicates that the CDKN1A gene locus, along with its methylation status and expression levels, plays a role in HF pathogenesis. The druggability analysis identified sodium salicylate and dicoumarol as potential therapeutic drugs. |
| 15 | **Limitations** | Discuss limitations of the study, taking into account the validity of the IV assumptions, other sources of potential bias, and imprecision. Discuss both direction and magnitude of any potential bias and any efforts to address them |  | …However, the study has certain limitations. The summary statistics for eQTL, mQTL, and pQTL come from varied sources, and the models used do not consistently account for confounders. While our study aligns with the core MR assumptions and includes pleiotropy analysis to reduce confounding, we cannot fully exclude its impact on the causal link between molecular traits and HF. The study's sample sizes for mQTL, GWAS data, and genetic variants tied to protein expression are limited, which may cause some HF-related genes to be missed. Additionally, the scarcity of GWAS datasets, especially those on cellular aging, restricts the bidirectional MR analysis of causality. Even with sensitivity analyses, pleiotropy assessment is still not precise, necessitating more individual-level data for refined stratified analysis. The study is based mainly on European population data, and while it was tested in East Asian groups, the specificity of genetic mutations restricts the wider applicability of the findings. Further functional experiments are required to validate these conclusions. |
| 16 | **Interpretation** |  |  |  |
|  | a) | Meaning: Give a cautious overall interpretation of results in the context of their limitations and in comparison with other studies |  | ……CDKN1A is a cyclin-dependent kinase inhibitor, has been known to regulate the cell cycle process [49]. Researchers have discovered that HF patients have an increased expression of CDKN1A. A crucial finding of our study confirms an increase in the expression level of CDKN1A in HF samples, similar to the findings published elsewhere [50]. Studies have indicated that CDKN1A may promote coronary heart disease by promoting chronic inflammation and sustained inflammatory states [51]. It has been reported that CDKN1A plays a significant role in inflammatory heart diseases because it is highly expressed in cardiomyocytes [52]. A possible link between DNAm, and the risk of HF is also revealed by our findings that DNAm in enhancer regions negatively regulates CDKN1A expression. There is a genetic variation near the CDKN1A gene associated with HF in previous GWAS studies [33], but whether this gene is causally related to the illness remains unclear. DNAm may be involved in genetic variation regulating gene expression in HF, which impacts its pathogenesis. It has been reported that EGR1 regulates CDKN1A expression, resulting in the senescence of cancer cells [53]. More research needs to be conducted to find out whether it plays a role in HF. |
|  | b) | Mechanism: Discuss underlying biological mechanisms that could drive a potential causal relationship between the investigated exposure and the outcome, and whether the gene-environment equivalence assumption is reasonable. Use causal language carefully, clarifying that IV estimates may provide causal effects only under certain assumptions |  | CDKN1A mechanism is complex across a wide range of cell types and stimulus environments. A number of studies have shown that CDKN1A- positive senescent cells exhibit a clear convergence, initially displaying high levels of CDKN1A due to damage, followed by a subsequent decrease [54]. Furthermore, the function of CDKN1A is influenced by the degree of DNA damage in the cell. When DNA damage is low, CDKN1A expression increases, slowing the cell division cycle and preventing apoptosis. In contrast, with higher levels of DNA damage, CDKN1A expression decreases, leading to apoptosis [55]. Additionally, our study observed that HF cardiomyocytes with CDKN1A+ show elevated levels of ubiquinone and other terpenoid-quinone biosynthesis, thiamine metabolism, and nicotinate and nicotinamide metabolism, suggesting metabolic discrepancies among HF cardiomyocytes with different CDKN1A genotypes. In addition to regulating fundamental processes such as cell cycle progression, apoptosis, and transcription, CDKN1A could also be responsible for the variation in phenotypes [56]. |
|  | c) | Clinical relevance: Discuss whether the results have clinical or public policy relevance, and to what extent they inform effect sizes of possible interventions |  | ……Biomarkers related to cardiac aging are a major focus in cardiovascular disease research. Compared to tissue markers and MRI[57], blood biomarkers provide advantages like simpler collection, reduced invasiveness, and the capacity for ongoing monitoring. The loci identified in this study shed new light on potential non-invasive biomarkers linked to HF. Current investigations into CDKN1A inhibitors for cancer therapies indicate promising possibilities for cardiovascular applications, which merit further investigation. More research is required to establish the safety and efficacy of these treatments in relation to HF. |
| 17 | **Generalizability** | Discuss the generalizability of the study results (a) to other populations, (b) across other exposure periods/timings, and (c) across other levels of exposure |  | This study has increased our understanding of how senescence may influence the biological mechanisms of HF. It has shown that methylation and gene expression related to CDKN1A may play a role in initiating senescence in HF cardiomyocytes. It may help identify potential new therapeutic targets for HF and advance fundamental research on the role of cellular senescence in the disease. |
|  | **OTHER INFORMATION** |  |  |  |
| 18 | **Funding** | Describe sources of funding and the role of funders in the present study and, if applicable, sources of funding for the databases and original study or studies on which the present study is based |  | This work was supported by the Henan Province Science and Technology Research Program（No.242102310528）. |
| 19 | **Data and data sharing** | Provide the data used to perform all analyses or report where and how the data can be accessed, and reference these sources in the article. Provide the statistical code needed to reproduce the results in the article, or report whether the code is publicly accessible and if so, where |  | Availability of data and material  The datasets presented in this study can be found in online repositories. The names of the repository/repositories and accession number(s) can be found in the article/ Supplementary Material. |
| 20 | **Conflicts of Interest** | All authors should declare all potential conflicts of interest |  | Declaration of Competing Interest  The authors have no competing interests to declare. |

This checklist is copyrighted by the Equator Network under the Creative Commons Attribution 3.0 Unported (CC BY 3.0) license.

1. Skrivankova VW, Richmond RC, Woolf BAR, Yarmolinsky J, Davies NM, Swanson SA, et al. Strengthening the Reporting of Observational Studies in Epidemiology using Mendelian Randomization (STROBE-MR) Statement. JAMA. 2021;under review.

2. Skrivankova VW, Richmond RC, Woolf BAR, Davies NM, Swanson SA, VanderWeele TJ, et al. Strengthening the Reporting of Observational Studies in Epidemiology using Mendelian Randomisation (STROBE-MR): Explanation and Elaboration. BMJ. 2021;375:n2233.
